# Supplementary material for: Gut metagenome associations with extensive digital health data in a volunteer-based Estonian microbiome cohort
Source: Nat Commun. 2022 Feb 15;13:869. doi: 10.1038/s41467-022-28464-9 (PMC8847343; doi:10.1038/s41467-022-28464-9)
Supplement: Supplementary file 1 — Supplementary Information [file 41467_2022_28464_MOESM1_ESM.pdf]

SUPPLEMENTARY INFORMATION

Gut metagenome associations with extensive digital health data in a volunteer-based Estonian microbiome cohort

Oliver Aasmets[1], Kertu Liis Krigul[1], Kreete Lüll[1], Andres Metspalu[1, 2, 3] and Elin Org[1*]

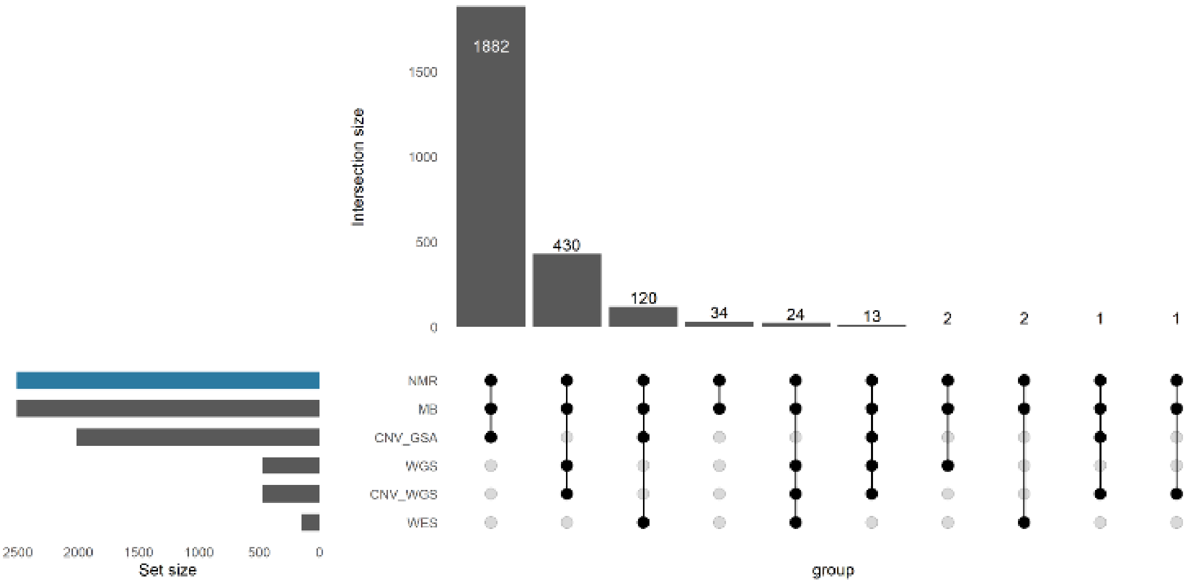

Supplementary Fig. 1. Omics data overlap in the Estonian Microbiome cohort. NMR metabolite data (highlighted in blue) will be available in 2022 from the same time points as MB collection. NMR, nuclear magnetic resonance; MB, microbiome; WES, whole-exome sequencing; WGS, whole-genome sequencing; CNV_GSA, copy number variation data from genotyped individuals; CNV_WGS, copy number variation data from individuals with whole-genome sequencing.

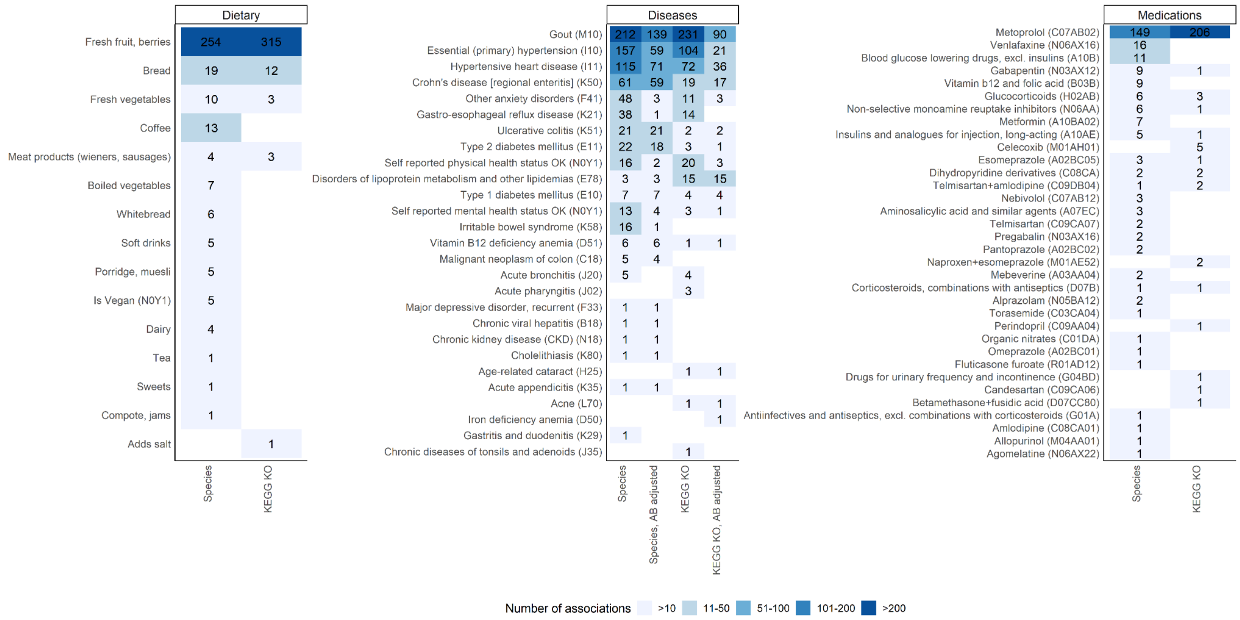

Supplementary Fig. 2. Summary of associations found by metagenome association analysis with microbial species and KOs. Abbreviations in the y-axis labels are the international classification of diseases-10 and anatomical therapeutic chemical codes for diagnoses and medications, respectively. AB, antibiotics; KEGG KO, Kyoto Encyclopedia of Genes and Genomes Orthology.

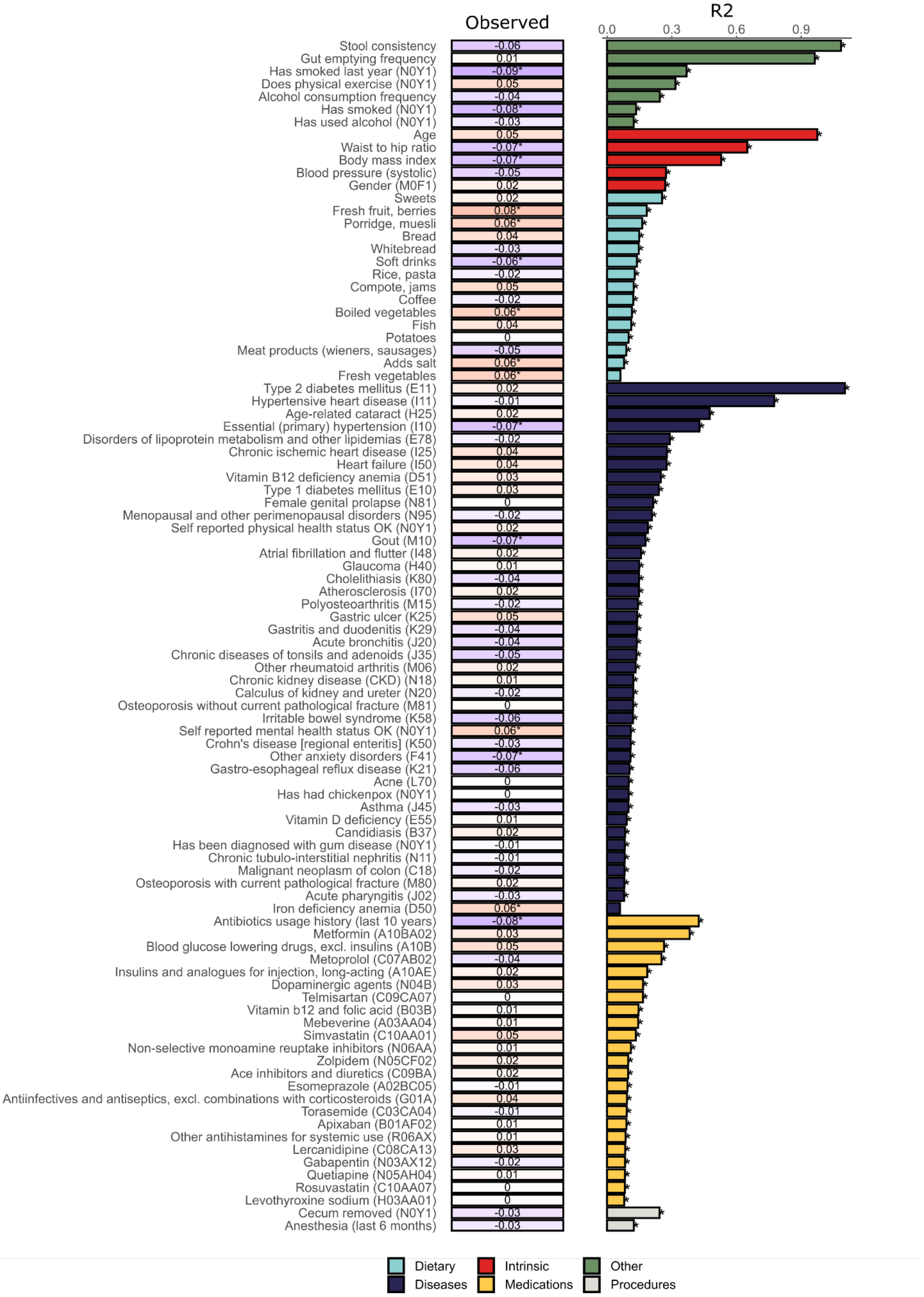

Observed
R2
0.0
0.3
0.6
0.9
Stool consistency -0.06
Gut emptying frequency 0.01
Has smoked last year (N0Y1) -0.09*
Does physical exercise (N0Y1) 0.05
Alcohol consumption frequency -0.04
Has smoked (N0Y1) -0.08*
Has used alcohol (N0Y1) -0.03
Age 0.05
Waist to hip ratio -0.07*
Body mass index -0.07*
Blood pressure (systolic) -0.05
Gender (M0F1) 0.02
Sweets 0.02
Fresh fruit, berries 0.08*
Porridge, muesli 0.06*
Bread 0.04
Whitebread -0.03
Soft drinks -0.06*
Rice, pasta -0.02
Compote, jams 0.05
Coffee -0.02
Boiled vegetables 0.06*
Fish 0.04
Potatoes 0
Meat products (wieners, sausages) -0.05
Adds salt 0.06*
Fresh vegetables 0.06*
Type 2 diabetes mellitus (E11) 0.02
Hypertensive heart disease (I11) -0.01
Age-related cataract (H25) 0.02
Essential (primary) hypertension (I10) -0.07*
Disorders of lipoprotein metabolism and other lipidemias (E78) -0.02
Chronic ischemic heart disease (I25) 0.04
Heart failure (I50) 0.04
Vitamin B12 deficiency anemia (D51) 0.03
Type 1 diabetes mellitus (E10) 0.03
Female genital prolapse (N81) 0
Menopausal and other perimenopausal disorders (N95) -0.02
Self reported physical health status OK (N0Y1) 0.02
Gout (M10) -0.07*
Atrial fibrillation and flutter (I48) 0.02
Glaucoma (H40) 0.01
Cholelithiasis (K80) -0.04
Atherosclerosis (I70) 0.02
Polyosteoarthritis (M15) -0.02
Gastric ulcer (K25) 0.05
Gastritis and duodenitis (K29) -0.04
Acute bronchitis (J20) -0.04
Chronic diseases of tonsils and adenoids (J35) -0.05
Other rheumatoid arthritis (M06) 0.02
Chronic kidney disease (CKD) (N18) 0.01
Calculus of kidney and ureter (N20) -0.02
Osteoporosis without current pathological fracture (M81) 0
Irritable bowel syndrome (K58) -0.06
Self reported mental health status OK (N0Y1) 0.06*
Crohn's disease [regional enteritis] (K50) -0.03
Other anxiety disorders (F41) -0.07*
Gastro-esophageal reflux disease (K21) -0.06
Acne (L70) 0
Has had chickenpox (N0Y1) 0
Asthma (J45) -0.03
Vitamin D deficiency (E55) 0.01
Candidiasis (B37) 0.02
Has been diagnosed with gum disease (N0Y1) -0.01
Chronic tubulo-interstitial nephritis (N11) -0.01
Malignant neoplasm of colon (C18) -0.02
Osteoporosis with current pathological fracture (M80) 0.02
Acute pharyngitis (J02) -0.03
Iron deficiency anemia (D50) 0.06*
Antibiotics usage history (last 10 years) -0.08*
Metformin (A10BA02) 0.03
Blood glucose lowering drugs, excl. insulins (A10B) 0.05
Metoprolol (C07AB02) -0.04
Insulins and analogues for injection, long-acting (A10AE) 0.02
Dopaminergic agents (N04B) 0.03
Telmisartan (C09CA07) 0
Vitamin b12 and folic acid (B03B) 0.01
Mebeverine (A03AA04) 0.01
Simvastatin (C10AA01) 0.05
Non-selective monoamine reuptake inhibitors (N06AA) 0.01
Zolpidem (N05CF02) 0.02
Ace inhibitors and diuretics (C09BA) 0.02
Esomeprazole (A02BC05) -0.01
Antiinfectives and antiseptics, excl. combinations with corticosteroids (G01A) 0.04
Torasemide (C03CA04) -0.01
Apixaban (B01AF02) 0.01
Other antihistamines for systemic use (R06AX) 0.01
Lercanidipine (C08CA13) 0.03
Gabapentin (N03AX12) -0.02
Quetiapine (N05AH04) 0.01
Rosuvastatin (C10AA07) 0
Levothyroxine sodium (H03AA01) 0
Cecum removed (N0Y1) -0.03
Anesthesia (last 6 months) -0.03
Dietary
Intrinsic
Other
Diseases
Medications
Procedures

Supplementary Fig. 3. Statistically significant associations with functional (KOs) alpha and beta diversity. The bar plot indicates the explained variance in the interindividual variation of microbial composition obtained by the permutational analysis of variance (based on the Euclidean distance on the CLR-transformed data). The heatmap shows the Spearman correlation coefficients of each factor with the observed KO richness. Blue indicates negative correlation, and red indicates positive correlation. Asterisks indicate associations with false discovery rate < 0.05. Abbreviations in the y-axis correspond to the international classification of diseases-10 codes for diagnoses and anatomical therapeutic chemical codes for medications.

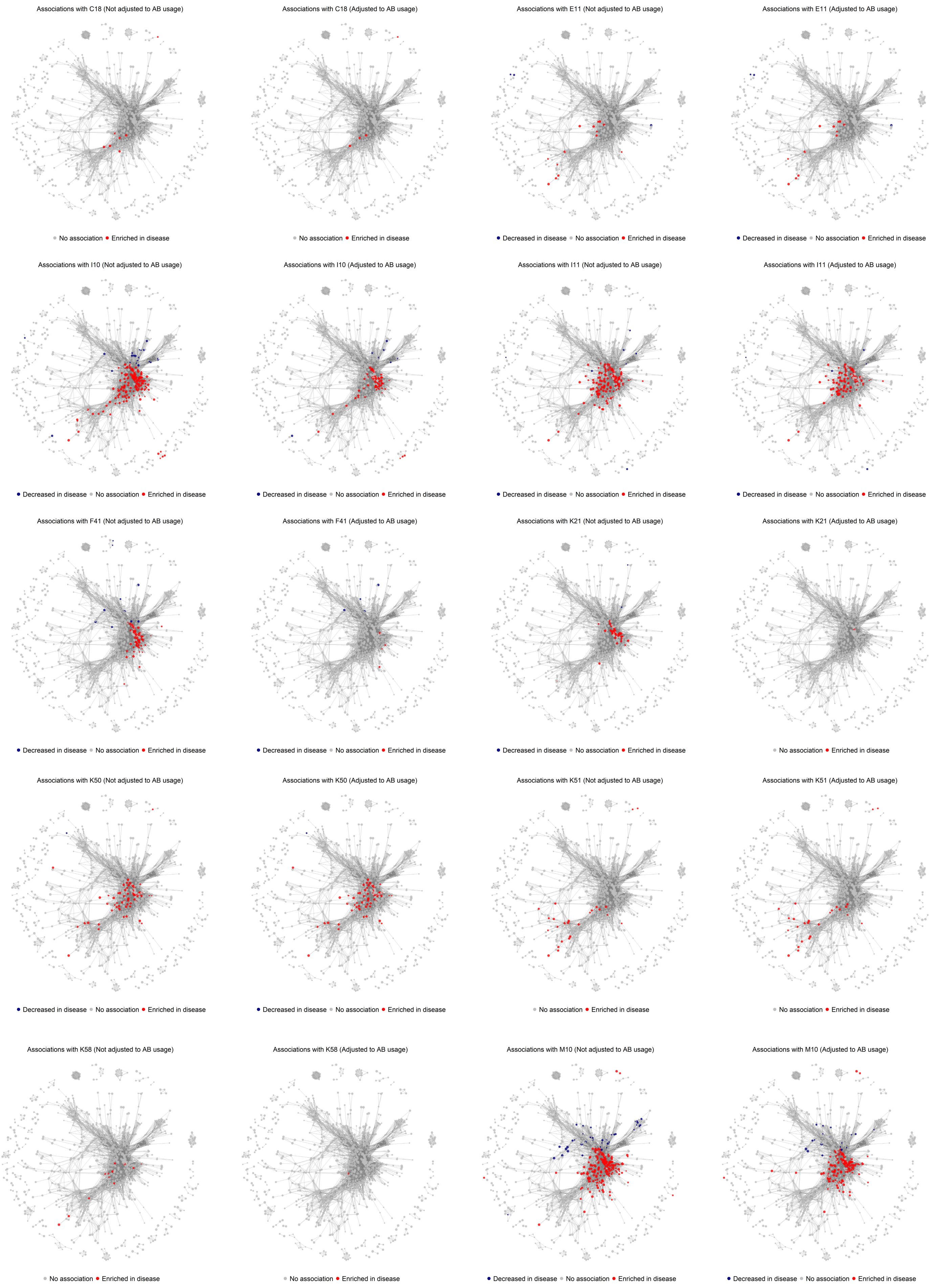

Associations with C18 (Not adjusted to AB usage)
No association Enriched in disease
Associations with C18 (Adjusted to AB usage)
No association Enriched in disease
Associations with E11 (Not adjusted to AB usage)
Decreased in disease No association Enriched in disease
Associations with E11 (Adjusted to AB usage)
Decreased in disease No association Enriched in disease
Associations with I10 (Not adjusted to AB usage)
Decreased in disease No association Enriched in disease
Associations with I10 (Adjusted to AB usage)
Decreased in disease No association Enriched in disease
Associations with I11 (Not adjusted to AB usage)
Decreased in disease No association Enriched in disease
Associations with I11 (Adjusted to AB usage)
Decreased in disease No association Enriched in disease
Associations with F41 (Not adjusted to AB usage)
Decreased in disease No association Enriched in disease
Associations with F41 (Adjusted to AB usage)
Decreased in disease No association Enriched in disease
Associations with K21 (Not adjusted to AB usage)
Decreased in disease No association Enriched in disease
Associations with K21 (Adjusted to AB usage)
No association Enriched in disease
Associations with K50 (Not adjusted to AB usage)
Decreased in disease No association Enriched in disease
Associations with K50 (Adjusted to AB usage)
Decreased in disease No association Enriched in disease
Associations with K51 (Not adjusted to AB usage)
No association Enriched in disease
Associations with K51 (Adjusted to AB usage)
No association Enriched in disease
Associations with K58 (Not adjusted to AB usage)
No association Enriched in disease
Associations with K58 (Adjusted to AB usage)
No association Enriched in disease
Associations with M10 (Not adjusted to AB usage)
Decreased in disease No association Enriched in disease
Associations with M10 (Adjusted to AB usage)
Decreased in disease No association Enriched in disease

Supplementary Fig. 4. Species co-occurrence networks depicting microbial associations with complex diseases before and after correction for long-term antibiotic (AB) usage. Each species (node) is colored according to its association with disease (blue corresponds to a negative association, and red corresponds to a positive association). Only statistically significant associations are shown (false discovery rate < 0.05). Abbreviations on top of the figures correspond to the international classification of diseases-10 codes for diagnoses.

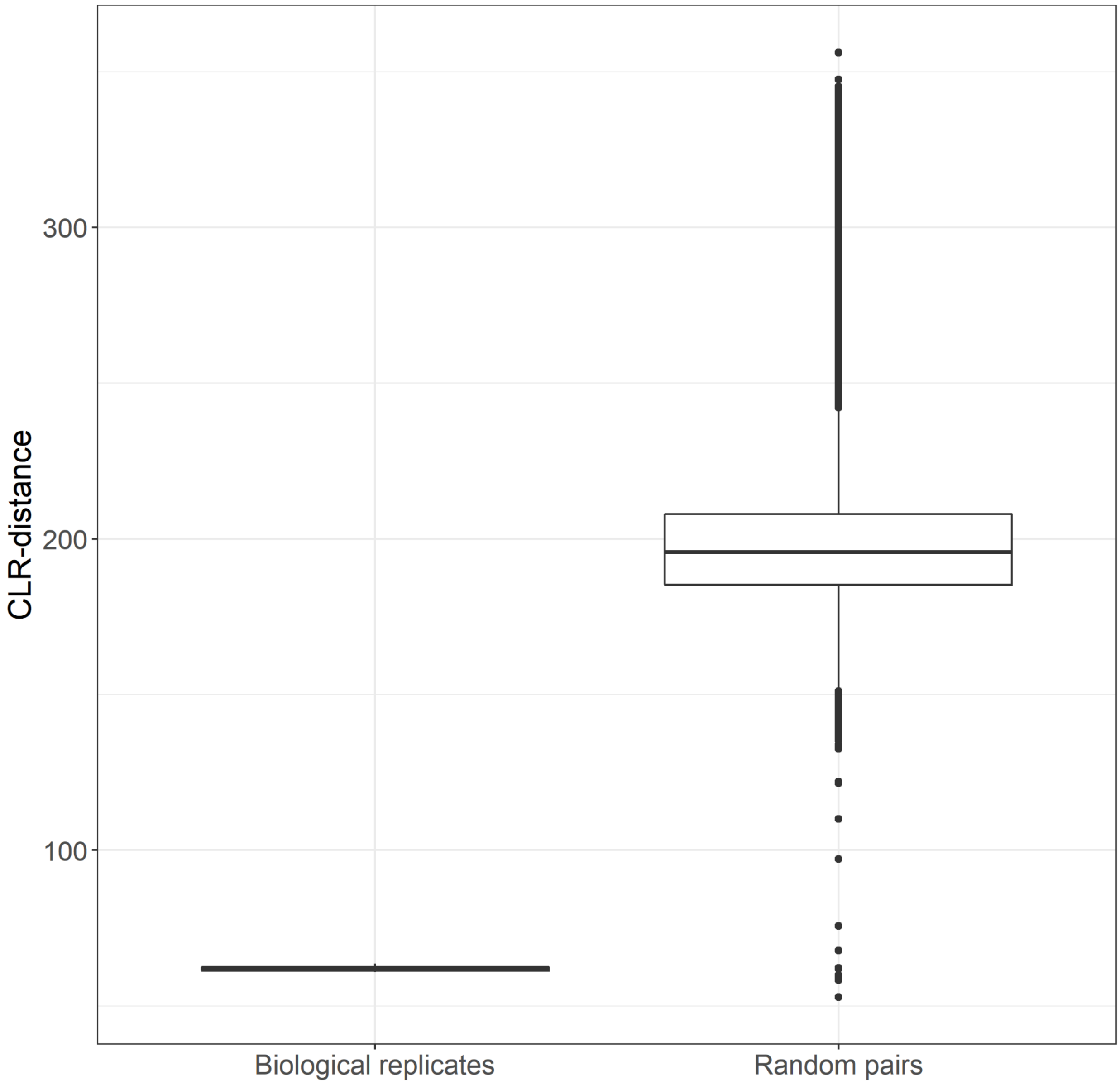

Supplementary Fig. 5. Similarity of the microbiome composition between biological replicates (n = 5) and pairs of unrelated samples (n = 2509) based on the Euclidean distance on the center log ratio (CLR)-transformed species-level microbiome profile. The central line, box and whiskers in boxplots represent the median, interquartile range (IQR), and 1.5 times the IQR, respectively.
