# Supplementary material for: Gut metagenome associations with extensive digital health data in a volunteer-based Estonian microbiome cohort
Source: Nat Commun. 2022 Feb 15;13:869. doi: 10.1038/s41467-022-28464-9 (PMC8847343; doi:10.1038/s41467-022-28464-9)
Supplement: Supplementary file 3 — Description of Additional Supplementary Files [file 41467_2022_28464_MOESM3_ESM.docx]

**Description of Additional Supplementary Files**

**File Name:** Supplementary Data 1

**Description:** Phenotype data summary statistics

**File Name:** Supplementary Data 2

**Description:** Summary statistics of kingdom, phylum and core genera relative abundances

**File Name:** Supplementary Data 3

**Description:** Alpha diversity analysis results (species and KEGG KO)

**File Name:** Supplementary Data 4

**Description:** Beta diversity analysis results (species and KEGG KO)

**File Name:** Supplementary Data 5

**Description:** MWAS univariate analysis results (number of cases analyzed, results for species and KEGG KOs)

**File Name:** Supplementary Data 6

**Description:** Summary table of identified associations

**File Name:** Supplementary Data 7

**Description:** Species and KEGG KOs in shared dysbiosis

**File Name:** Supplementary Data 8

**Description:** Mean AUROC values for predictive modelling

**File Name:** Supplementary Data 9

**Description:** Number of cases for all three-digit ICD10 codes in the EstMB cohort
