# Supplementary material for: Gut metagenome associations with extensive digital health data in a volunteer-based Estonian microbiome cohort
Source: Nat Commun. 2022 Feb 15;13:869. doi: 10.1038/s41467-022-28464-9 (PMC8847343; doi:10.1038/s41467-022-28464-9)
Supplement: Supplementary file 13 — Reporting Summary [file 41467_2022_28464_MOESM13_ESM.pdf]

nature portfolio

Corresponding author(s): Elin Org

Last updated by author(s): 20.01.2022

Reporting Summary

Nature Portfolio wishes to improve the reproducibility of the work that we publish. This form provides structure for consistency and transparency in reporting. For further information on Nature Portfolio policies, see our Editorial Policies and the Editorial Policy Checklist.

Statistics

For all statistical analyses, confirm that the following items are present in the figure legend, table legend, main text, or Methods section.

n/a	Confirmed	
☐	☒	The exact sample size (*n*) for each experimental group/condition, given as a discrete number and unit of measurement
☐	☒	A statement on whether measurements were taken from distinct samples or whether the same sample was measured repeatedly
☐	☒	The statistical test(s) used AND whether they are one- or two-sided<br>*Only common tests should be described solely by name; describe more complex techniques in the Methods section.*
☐	☒	A description of all covariates tested
☐	☒	A description of any assumptions or corrections, such as tests of normality and adjustment for multiple comparisons
☐	☒	A full description of the statistical parameters including central tendency (e.g. means) or other basic estimates (e.g. regression coefficient) AND variation (e.g. standard deviation) or associated estimates of uncertainty (e.g. confidence intervals)
☐	☒	For null hypothesis testing, the test statistic (e.g. *F*, *t*, *r*) with confidence intervals, effect sizes, degrees of freedom and *P* value noted<br>*Give P values as exact values whenever suitable.*
☒	☐	For Bayesian analysis, information on the choice of priors and Markov chain Monte Carlo settings
☒	☐	For hierarchical and complex designs, identification of the appropriate level for tests and full reporting of outcomes
☐	☒	Estimates of effect sizes (e.g. Cohen's *d*, Pearson's *r*), indicating how they were calculated

Our web collection on statistics for biologists contains articles on many of the points above.

Software and code

Policy information about availability of computer code

Data collection	Information on how phenotype data was collected is available in Methods. No specific software was used for phenotype data collection. Raw sequencing data were deposited in the European Genome Archive (EGA) with accession number: EGAD00001008448
Data analysis	Published software applications, libraries and code used: SOAP2.21 (for host read removal, and clean data mapping), SOAPdenovo v.2.04 (for metagenomic assembly), MetaGeneMark v.3.38 (for gene prediction), CD-HIT v.4.6 (for dereplication of the genes and defining representative gene), SoapAligner v.2.21(mapping to gene catalogues and quantifying the genes), DIAMOND v.0.9.9.110 (for taxonomic assignment). Custom R code (for all downstream statistical analyses) which utilizes libraries vegan (v2.5.6), ALDEx2(v.1.18.0), tidymodels (v0.1.1), glmnet (v3.0-2) and SPIEC-EASI (v1.1.0) is is available at https://doi.org/10.5281/zenodo.5767071.

For manuscripts utilizing custom algorithms or software that are central to the research but not yet described in published literature, software must be made available to editors and reviewers. We strongly encourage code deposition in a community repository (e.g. GitHub). See the Nature Portfolio guidelines for submitting code & software for further information.

Data

Policy information about availability of data

All manuscripts must include a data availability statement. This statement should provide the following information, where applicable:

- Accession codes, unique identifiers, or web links for publicly available datasets
- A description of any restrictions on data availability
- For clinical datasets or third party data, please ensure that the statement adheres to our policy

The metagenomic data generated in this study have been deposited in the European Genome-Phenome Archive database (https://www.ebi.ac.uk/ega/) under accession code EGAS00001008448 [https://ega-archive.org/datasets/EGAD00001008448]. The phenotype data contain sensitive information from healthcare

registers and they are available under restricted access through the Estonian biobank upon submission of a research plan and signing a data transfer agreement. All data access to the Estonian Biobank must follow the informed consent regulations of the Estonian Committee on Bioethics and Human Research, which are clearly described in the Data Access section at https://genomics.ut.ee/en/content/estonian-biobank. A preliminary request for raw metagenome and phenotype data must first be submitted via the e-mail address releases@ut.ee. Used databases are NCBI nonredundant (NCBI nr) database 201810 (https://www.ncbi.nlm.nih.gov/blast/db/) and KEGG (https://www.kegg.jp/).

Field-specific reporting

Please select the one below that is the best fit for your research. If you are not sure, read the appropriate sections before making your selection.

[x] Life sciences [ ] Behavioural & social sciences [ ] Ecological, evolutionary & environmental sciences

For a reference copy of the document with all sections, see nature.com/documents/nr-reporting-summary-flat.pdf

Life sciences study design

All studies must disclose on these points even when the disclosure is negative.

Sample size	In total, 2509 fecal samples were collected from 2017-2019 from the Estonian Biobank participants. No sample size calculations were performed, as the primary objective of the study was exploratory in order to uncover the microbiome associations with different health parameters in Estonian population. The sample size was determined by the availability of resources.
Data exclusions	Of 2509 volunteers, samples from 3 subjects were excluded as their total sample read counts were considered outliers compared to the other samples, yielding n=2506 available for all downstream analyses. For univariate analysis and predictive modeling, patients who had recently (within 6 months) used antibiotics were excluded, resulting in 2024 samples. Further 73 were excluded, as only 1951 participants had the full covariate information available (age, BMI, gender and Bristol stool scale). The exclusion criteria were not pre-established.
Replication	Due to the nature of the article being exploratory, no direct replication was done. However, we were successful at replicating the associations between microbial diversity and all of the major factors reported in microbiome studies, such as stool consistency, gut emptying frequency, BMI and age.
Randomization	Participants were not randomly allocated to experimental groups. The study included a random subsample of the volunteer-based Estonian Biobank participants, which includes the adult population across Estonia. Factors known to influence microbiome variation were recorded using participants' self-reported answers to questionnaire (Bristol stool scale), data collected by a trained personnel (BMI, age, gender), and national electronic health registries (diseases and medication usage data from Estonian Health Insurance Fund, Estonian Cancer Registry, University of Tartu Clinic, and North Estonia Medical Centre). Statistical analysis were performed considering coufounding factors.
Blinding	Issue of blinding was not relevant, as the study had no experimental group allocation.

Reporting for specific materials, systems and methods

We require information from authors about some types of materials, experimental systems and methods used in many studies. Here, indicate whether each material, system or method listed is relevant to your study. If you are not sure if a list item applies to your research, read the appropriate section before selecting a response.

Materials & experimental systems

n/a	Involved in the study	
[x]	[ ]	Antibodies
[x]	[ ]	Eukaryotic cell lines
[x]	[ ]	Palaeontology and archaeology
[x]	[ ]	Animals and other organisms
[ ]	[x]	Human research participants
[x]	[ ]	Clinical data
[x]	[ ]	Dual use research of concern

Methods

n/a	Involved in the study	
[x]	[ ]	ChIP-seq
[x]	[ ]	Flow cytometry
[x]	[ ]	MRI-based neuroimaging

Human research participants

Policy information about studies involving human research participants

Population characteristics	The study included a subsample of the volunteer-based Estonian Biobank participants, which includes the adult population (≥18 years old) across Estonia. We recruited in total of 2509 subjects, of whom 70.3% were women (n = 1,764) and 29.7% were men (n = 745), with mean age of 50 years. No exclusion criteria were applied before the sample collection. The vast majority of the participants are of Estonian origin (98.4%).
Recruitment	Participants from the Estonian Biobank were invited to the study using electronic invitation or letter in mail. Participants were recruited from all over Estonia. Selection bias could have been induced through the recruitment. There are more females in the EstMB cohort (70% of the participants), reflecting the structure in the Estonian biobank (2/3 volunteers are female), but not reflecting the structure of Estonian population. As the cohort is volunteer-based and no compensation is paid, the study might attract people who are more interested about their health in general. Additionally, as the focus of the study is the gut microbiome, the participants might be more interested in their gut health and thus might have more intestinal problems than general population.
Ethics oversight	This study was approved by the Research Ethics Committee of the University of Tartu (approval No. 266/T10) and by the Estonian Committee on Bioethics and Human Research (Estonian Ministry of Social Affairs; approval No. 1.1-12/17). All participants have joined the Estonian Biobank on a voluntary basis and have signed a broad consent form, which allows to receive participant's personal and health data from national registries and databases. Rights of gene donors are regulated by Human Genes Research Act (HGRA) § 9 – Voluntary nature of gene donation (https://www.riigiteataja.ee/en/eli/ee/531102013003/consolide/current).

Note that full information on the approval of the study protocol must also be provided in the manuscript.
